# Survey dataset on the performance of combined process of coagulation and ceramic membrane for the removal of reactive black19

**DOI:** 10.1016/j.dib.2018.06.114

**Published:** 2018-07-03

**Authors:** Ali Jafari, Bahram Kamarehi, Akram Ghasemi

**Affiliations:** a Razi Herbal Medicines Research Center, Lorestan University of Medical Sciences, Khorramabad, Iran; b Department Of Environmental Health Engineering, Faculty of Health and Nutrition, Lorestan University of medical sciences, Khorramabad, Iran

**Keywords:** Alum, Ceramic membrane, Chemical coagulants, Ferric chloride, Reactive black19

## Abstract

The aim of data article was to evaluate effectiveness of the combined process of coagulation, flocculation and filtration to remove reactive black 19 from solution aqueous. In this data article, alum and ferric chloride were selected as a coagulant for instability of color before ceramic membrane. All experiments were performed in batch conditions. The results showed that more than half of the ceramic membrane is made of silica. The BET surface area of the ceramic membrane was 1.877 m^2^ g^−1^. The size of RB19, alum, and ferric chloride was 996.2 mm, 1216 mm, and 86.26 mm, respectively. Also, the zeta potential of RB19, alum, and ferric chloride was 20.7 mV, 1.59 mV, and 34.7 mV, respectively. The findings showed that the best pH to remove the RB 19 by alum and ferric chloride was less than 7. With increasing concentrations of alum and chlorine ferric the removal of RB 19 increased. For RB19 concentration of 10 mg l^−1^, with increasing time from 5 to 15 min, the removal efficiency for alum and ferric were 61–63% and 82–87%, respectively.

**Specifications Table**

**Table:** 

Subject area	*Water Chemistry*
More specific subject area	*Water Treatment*
Type of data	*Table, figure*
How data was acquired	*spectrophotometer (UV-UVIS, 594 nm)*
Data format	*Raw, analyzed,*
Experimental Factors	*All experiments were performed in batch conditions. The main variables were investigated such as pH, color concentration, coagulation concentration, mixing speed, filtration time. In this data article, the coagulations of alum and ferric chloride were used as coagulant. The final concentration of RB 19 was determined by a spectrophotometer (UV-UVIS, 594 nm)*
Experimental features	*To determine the structural properties of color, fluke and ceramic membrane by X-ray fluorescence (XRF) spectrometer, Fourier Transform Infrared spectroscopy spectra (FTIR) (Spectrum Two model, PerkinElmer Company), X-ray Diffraction (X’ Pert Pro model, Panalytical Company), Energy Dispersive X-ray Spectroscopy, Field Emission Scanning Electron Microscopy (SIGMA VP-500 model, ZEISS Company), and BET surface area and total pore volumes of the samples (BElSORP Mini model, Microtrac Bel Corp) were determined from nitrogen adsorption isotherms at 77 K.*
Data source location	Khorramabad city, Lorestan province, west of Iran
Data accessibility	*Data are included in this article*

**Value of the data**

● The hybrid process of coagulation and flocculation was applied to remove reactive black 19 from solution aqueous.
● The data was obtained by sufficient experiments and repetition. The data are useful for developing the same processes and applying for larger scale process.
● This data article shows the capability of the hybrid process of coagulation and flocculation to remove colored effluent.
● The data obtained from this study showed that this combined process could be of interest to environmental authorities.

## 1 Data

In this data article, alum and ferric chloride coagulants as pretreatment step had been suggested to reduce reactive black (RB) color before further treated by ceramic membrane process. The study, RB 19 investigated.

## 2 Experimental design, materials, and methods

**Fig. 1:**
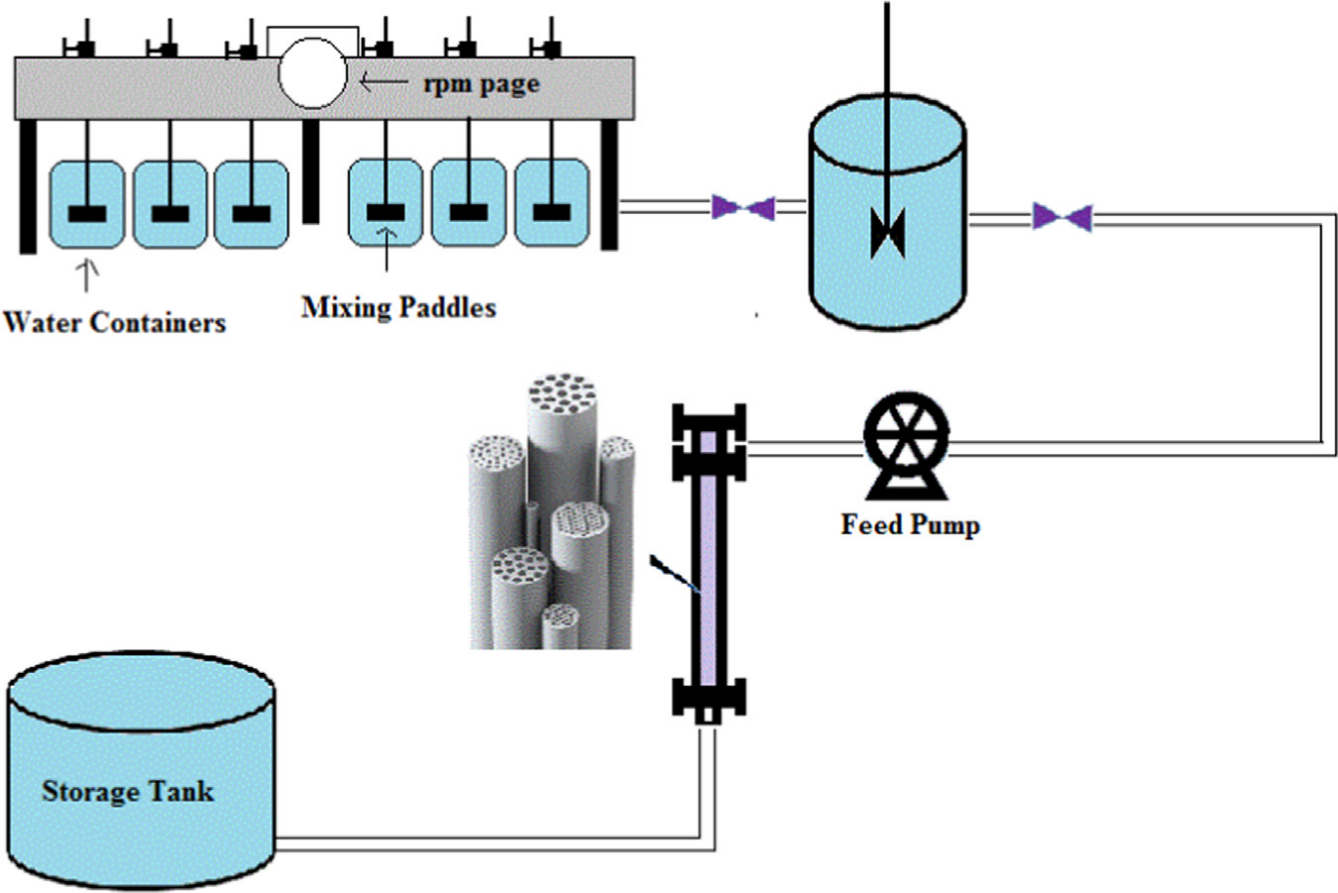
A view of the used pilot to remove RB 19.

All experiments were performed in batch conditions. The main variables were investigated such as pH, color concentration, coagulation concentration, mixing speed, filtration time. In this data article, the coagulations of alum and ferric chloride were used as coagulant. The final concentration of RB 19 was determined by a spectrophotometer (UV–vis, 594 nm). At first for color measurement, calibration curves were drawn (*Y* = 44.603, *R*^2^ = 0.9993). Fig. 1 shows used pilot for removal of RB 19. In the Jar-Test, the volume of color solution was 500 ml. Rapid mix speed and contact time were 120 rpm and 1 min, respectively. Slow mix speed and contact time were 20, 30, 40 rpm and 20 min, respectively. Alum and ferric chloride coagulants were added at various dosages with a fixed color concentration in different pH values.

**Fig. 2:**
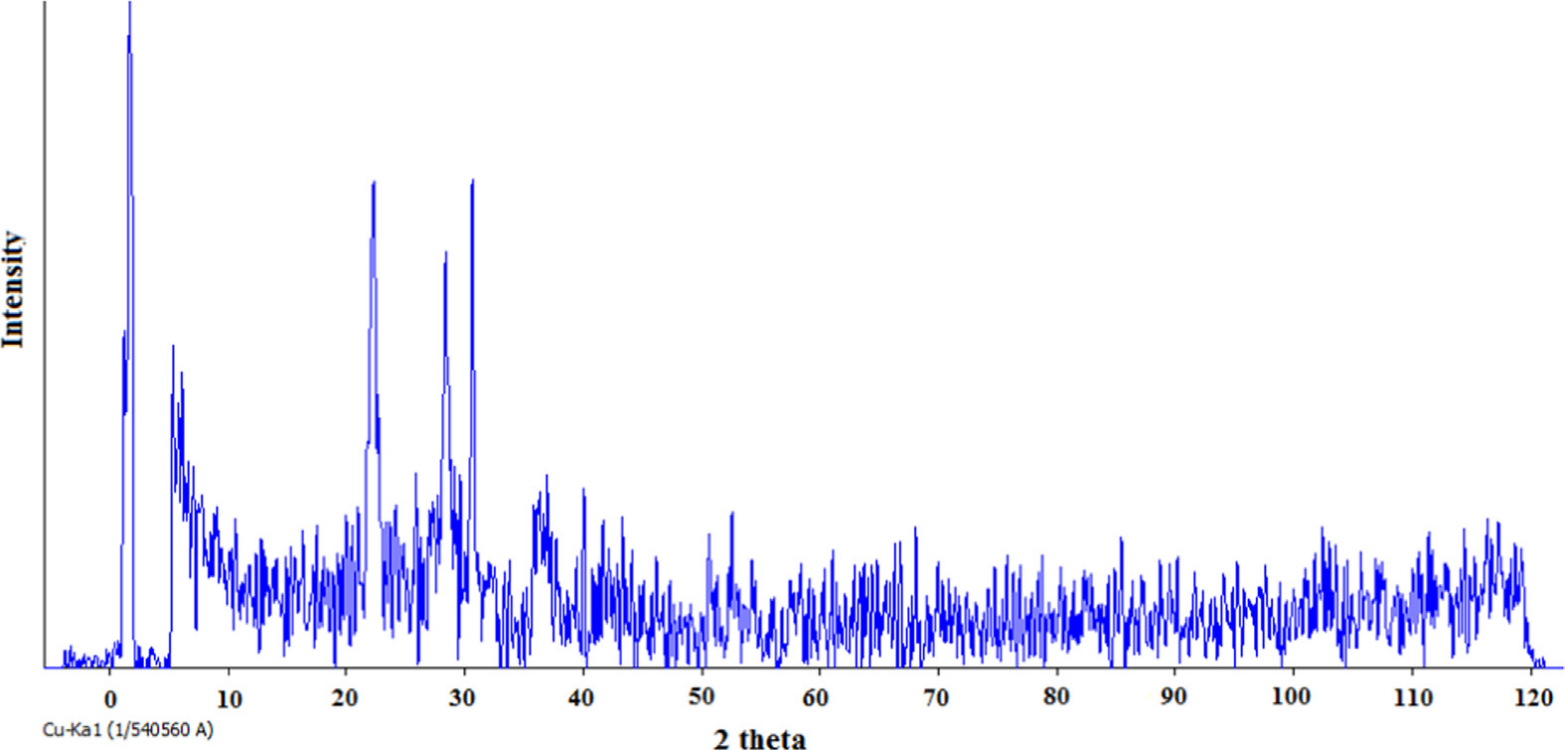
X-ray diffraction analysis spectra of the ceramic membrane.

**Fig. 3:**
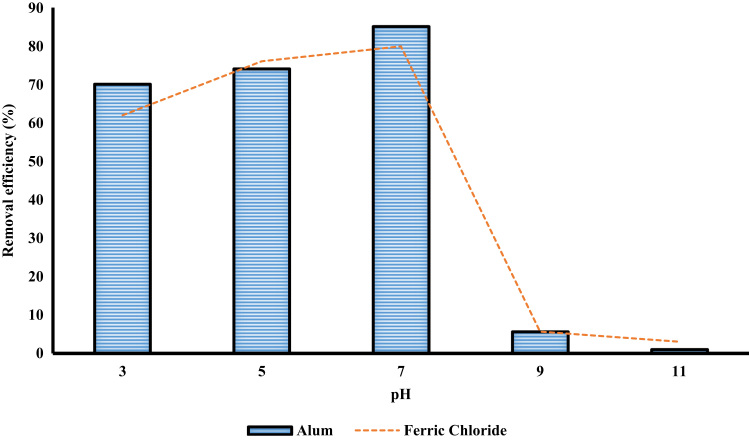
The effect of pH changes on color removal of RB19 by coagulants ( alum and ferric chloride, and color concentration 30, 30, and 30 mg/l, respectively).

**Fig. 4:**
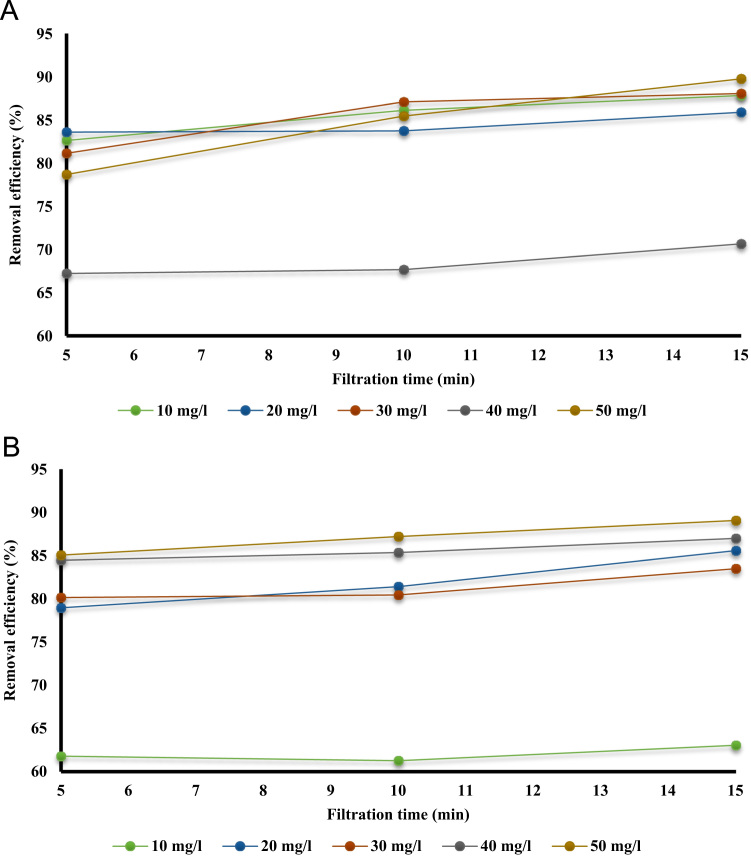
The effects of coagulant dosage on remove of color A. ferric chloride, B. alum.

**Fig. 5:**
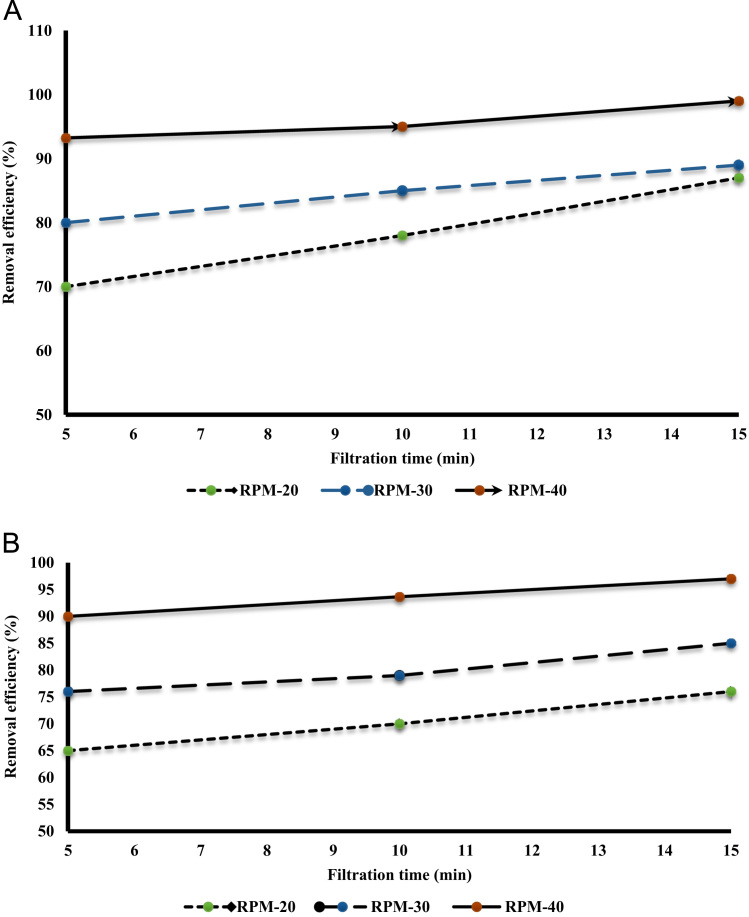
Changes of combined efficiency in various rpm A. ferric chloride, B. alum.

**Fig. 6:**
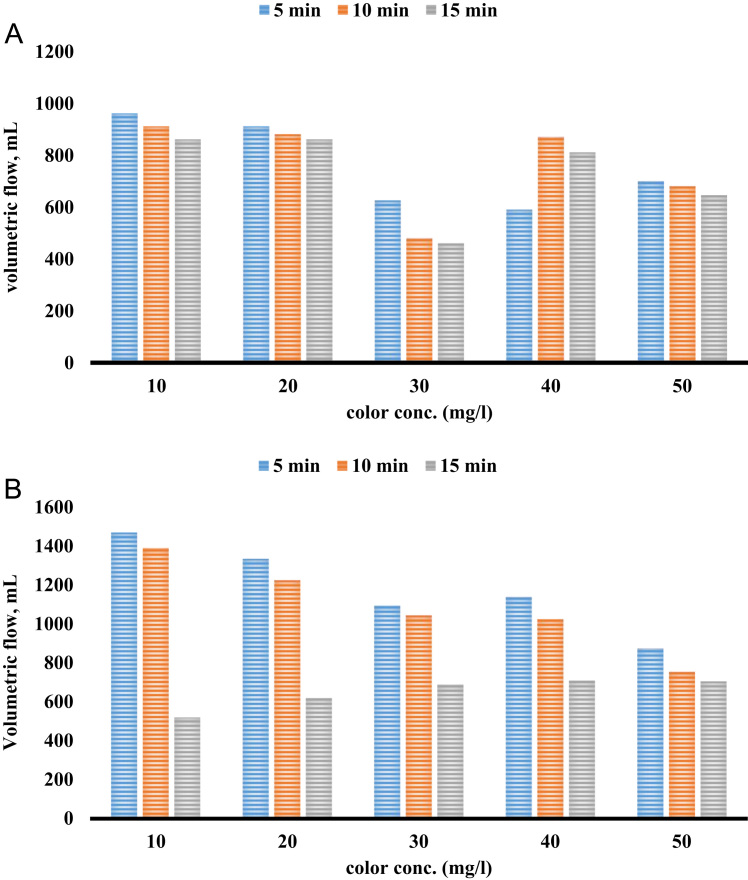
A. ferric chloride + ceramic filter, B. alum + ceramic filter.

Table 1 shows the constituent elements of the ceramic membrane. More than half of the membrane is made of silica (SiO_2_ ~ 57%). Fig. 2 shows X-ray diffraction analysis of ceramic membrane. The BET surface area of the ceramic membrane was 1.877 m^2^ g^−1^. Some properties of the ceramic membrane is shown Table 2. To examine for the morphology of ceramic membrane and flock on the filter, scanning electron microscopy was used. The size of RB19, alum, and ferric chloride was 996.2, 1216, and 86.26 mm, respectively. To measure the surface charge density of the particles, zeta potential test was used. The zeta potential of RB19, alum, and ferric chloride was 20.7, 1.59, and 34.7 mV, respectively. To verify the existence of different functional groups present in RB19 and the ceramic membrane. The FTIR analysis was carried out in the wavelengths 600–3500 cm^−1^. Functional groups in the ceramic membrane were C–S (630–790 cm^−1^), C = S (1000–1250 cm^−1^), C = N (1610–1680 cm^−1^), C = C (2100–2250 cm^−1^), C–H (3000–3100 cm^−1^), and O-H (3100–3650 cm^−1^). Fig. 3 shows the performance of two coagulants at different pH values. The findings showed that the best pH to remove the RB 19 by alum as coagulant was less than 7. At pH = 7, the color removal was almost 85%. Fig. 4 shows the results of removal of colors with different dosage of alum and chlorine ferric coagulants. The results showed that with increasing concentrations of chlorine ferric the removal efficiency of RB19 increased. The highest removal efficiency of color was observed at 50 mg l^−1^. Table 3 shows the changes of removal efficiency with color concentration. With the increasing color concentration in constant dosage, removal efficiency gradually reduced. To ferric coagulant with increasing RB19 concentration from 10 to 50 mg l^−1^, the removal efficiency reduced. Fig. 5 shows the removal of color in with different mixing rates of ferric chlorine and alum coagulant. The results showed that with the increase of slow mixing rate, the color removal increased. Fig. 6 show permeate flux of combined process of coagulation (ferric chloride and alum) and the ceramic filter. The results showed that the permeate flux declines by the time operation [1], [2], [3], [4], [5], [6], [7], [8], [9], [10], [11], [12], [13], [14], [15], [16], [17], [18].

**Table 1:** The constituent elements of the ceramic membrane.

**Components**	**%**	**Components**	**%**
SiO_2_	56.81	P_2_O_5_	0.75
Al_2_O_3_	16.87	SO_3_	Less than 0.1
CaO	16.7	TiO_2_	0.15
MgO	1.63	Na_2_O	0.59
Fe_2_O_3_	2.04	K_2_O	0.74
MnO	Less than 0.1	V_2_O_5_	Less than 0.1

**Table 2:** Some properties of ceramic membrane for nitrogen adsorption-desorption experiments at 77 k.

**Sample**	**SA**_**BET**_**(m**^**2**^**g**^**−1**^**)**	**Total pore volume (cm**^**3**^**g**^**−1**^**)**	**Mean pore diameter (nm)**	**Special capacity**
Ceramic membrane	1.877	3.8135	128.43	45.085

**Table 3:** Changes of combined efficiency in various concentrations of RB19.

Coagulant type	Filtration time (min)	Color concentration (mg/l)				
		**10**	**20**	**30**	**40**	**50**
	5	82.60	83.57	81.12	67.22	78.66
**Ferric chloride**	10	86.10	83.72	87.07	67.66	85.43
	15	87.81	85.88	88.03	70.64	89.74
	5	61.79	78.96	80.15	84.46	85.06
**Alum**	10	61.27	81.42	80.45	85.36	87.21
	15	63.05	85.58	83.50	86.99	89.07
