# Supplementary material for: Survey dataset on the performance of combined process of coagulation and ceramic membrane for the removal of reactive black19
Source: Data Brief. 2018 Jul 3;19:2015–22. doi: 10.1016/j.dib.2018.06.114 (PMC6141443; doi:10.1016/j.dib.2018.06.114)
Supplement: Supplementary file 1 — Transparency document. [file mmc1.docx]

Dear Professor,

Editor of **Data in Brief**

The authors of this article declare that they have no conflict of interests.
